# Media photo‐degradation in pharmaceutical biotechnology – impact of ambient light on media quality, cell physiology, and IgG production in CHO cultures

**DOI:** 10.1002/jctb.5643

**Published:** 2018-06-01

**Authors:** Lukas Neutsch, Paul Kroll, Matthias Brunner, Alexander Pansy, Michael Kovar, Christoph Herwig, Tobias Klein

**Affiliations:** ^1^ Research Division Biochemical Engineering Vienna University of Technology, Institute of Chemical Engineering Vienna Austria; ^2^ CD Laboratory on Mechanistic and Physiological Methods for Improved Bioprocesses Vienna University of Technology Vienna Austria

**Keywords:** analytical biotechnology, bioprocess monitoring, antibodies, flow cytometry

## Abstract

**BACKGROUND:**

Many vital components in bioprocess media are prone to photo‐conversion or photo‐degradation upon exposure to ambient light, with severe negative consequences for biomass yield and overall productivity. However, there is only limited awareness of light irradiation as a potential risk factor when working in transparent glass bioreactors, storage vessels or disposable bag systems. The chemical complexity of most media renders a root‐cause analysis difficult. This study investigated in a novel, holistic approach how light‐induced changes in media composition relate to alterations in radical burden, cell physiology, morphology, and product formation in industrial Chinese hamster ovary (CHO) bioprocesses.

**RESULTS:**

Two media formulations from proprietary and commercial sources were tested in a pre‐hoc light exposure scenario prior to cultivation. Using fluorescence excitation/emission (EEM) matrix spectroscopy, a photo‐sensitization of riboflavin was identified as a likely cause for drastically decreased IgG titers (up to −80%) and specific growth rates (−50% to −90%). Up to three‐fold higher radical levels were observed in photo‐degraded medium. On the biological side, this resulted in significant changes in cell morphology and aberrations in the normal IgG biosynthesis/secretion pathway.

**CONCLUSION:**

These findings clearly illustrate the underrated impact of room light after only short periods of exposure, occurring accidentally or knowingly during bioprocess development and scale‐ up. The detrimental effects, which may share a common mechanistic cause at the molecular level, correlate well with changes in spectroscopic properties. This offers new perspectives for online monitoring concepts, and improved detectability of such effects in future. © 2018 The Authors. *Journal of Chemical Technology & Biotechnology* published by JohnWiley & Sons Ltd on behalf of Society of Chemical Industry.

**ABBREVIATIONS**
BSA: bovine serum albumin
CDPM: chemically defined proprietary medium
CHO: Chinese hamster ovary
DAPI: 4′,6‐diamidino‐2‐phenylindole
DCF: 2′,7′‐dichlorofluorescein
DCFH: 2′,7′‐dichlorodihydrofluorescein
DMEM: Dulbecco's Modified Eagle Medium
EDTA: ethylenediaminetetraacetic acid
EEM: excitation/emission matrix spectroscopy
ER: endoplasmic reticulum
ex/em: excitation/emission
FCS: fetal calf serum
FITC: fluorescein isothiocyanate
HEPES: 4‐(2‐hydroxyethyl)‐1‐piperazineethanesulfonic acid
HPLC/MS: high performance liquid chromatography /mass spectrometry coupling
IgG: immunoglobulin G
LDH: lactate dehydrogenase
LET: light exposure time
LmC: lumichrome
MWP: microwell plate
PBS: phosphate buffered saline
qP: cell‐specific productivity
Rf: riboflavin
RNS: reactive nitrogen species
ROS: reactive oxygen species
RPMI: Roswell Park Memorial Institute medium
Trp: tryptophan
Tyr: tyrosine
UV: ultraviolet
VCC: viable cell concentration
VCC_max_: maximum viable cell concentration
WGA: wheat germ agglutinin

## INTRODUCTION

It is well known that several vital components in cell culture media are sensitive to ambient light, and can undergo photo‐chemical conversion within hours or even minutes.^1^, ^2^ Riboflavin (Rf), tryptophan (Trp) and tyrosine (Tyr) are among the most prominent examples of substances involved in light‐induced degradation processes and in the photosensitized generation of reactive oxygen species.^3^, ^4^, ^5^, ^6^, ^7^, ^8^ While effects of this kind have first been described more than four decades ago, the mechanistic basis of medium deterioration and especially the resultant impact on bioprocess performance, culture physiology and productivity is still poorly understood.

In industrial‐scale manufacture, media preparation and subsequent processing steps are usually carried out in steel vessels that provide near‐complete light protection. However, this is not necessarily the case in the earlier phases of process development and optimization. Microscale bioreactor platforms for high‐throughput screening typically use light‐transparent, disposable cuvettes, and initial process optimization studies are often performed in glass vessels at the 1–3 L scale. Moreover, there is an increasing trend towards single‐use systems especially in the mid‐scale (50–2000 L) production of high value‐added compounds, which goes along with an increasing use of transparent or semi‐transparent polymer bags.^9^ The light‐associated impact on overall culture performance may differ between setups (depending on the degree of light exposure), and hence may lead to unexplained bias during scale‐up operations. A recent study gave alarming proof of the importance of such phenomena by showing that only slight changes in the lighting conditions can lead to ample shifts in the configuration of the expressed monoclonal antibody product.^10^

Since 1996, photostability testing of finished products is an integral part of the ICH Q1B guidelines, but no recommendations exist on how to detect and quantify photo‐damaging effects during (fermentation) or even before (medium preparation and storage) the production phase.^11^ Due to cost/time considerations, it is in most cases not practically feasible to perform a complete assessment of all chemical species found in a cell culture medium (e.g. via HPLC/MS). Routine application in quality control calls for a rapid and easy‐to‐conduct fingerprinting method that can assess the relevant changes with high accuracy but without the need for complex sample workup or expensive equipment. Fortunately, the same molecular characteristics that render a compound susceptible to photo‐induced damage also render it an attractive target for spectroscopic analysis. Using its fluorescence properties, subtle changes in the configuration of a molecule can be monitored with high sensitivity for the typical concentration ranges found in media.^5^, ^12^

In this study, a holistic approach was used to investigate the functional link from medium light exposure, changes in media spectral properties, ROS/RNS formation, culture physiology and product formation in CHO cells expressing a high‐value pharmaceutical antibody drug. By comparing a defined, proprietary (CDPM) and a serum‐supplemented commercial medium (DMEM) under standardized illumination and growth conditions, it was demonstrated that the observable light‐induced effects may be rather general in nature and not confined to one specific mix of components. It is shown that fluorescence‐spectroscopic analysis allows for facile and specific assessment of different photosensitized processes in the media, and which of the processes may be of particular relevance for culture physiology and productivity. Results reveal a clear, performance‐compromising influence of light irradiation, and highlight the need to check for unintended media photo‐damage during bioprocess development and scale‐up.

## MATERIALS AND METHODS

### Medium light irradiation

Inadvertent or knowingly tolerated exposure of media to ambient light is most likely to happen during preparation or cultivation on the laboratory bench, and less likely during storage in freezers or cold rooms where the lights are proven to be off and light exposure rates are monitored. The light irradiation protocol for this study was performed at room temperature (22 ± 1°C) in order to assess potential additive effects of light and temperature‐mediated degradation. Samples for dark control were kept under the same temperature conditions but shielded from light to allow for direct comparison. Media samples were aliquoted in airtight 500 mL laboratory glass bottles (Duran^®^), placed on an orbital shaking platform and exposed to a commercially available standard light source (four E27 energy‐saving daylight bulbs; 20 W, 6500 K, 1215 lm) under a closed fume hood. A fixed setup with defined illumination distance was used throughout the study to ensure reproducible conditions. For each experiment, the medium was prepared as a single batch and stored at 4°C in the dark prior to portioning in bottles and initiating the light exposure phase (0–96 h). After light exposure, media were immediately used for batch cultivations without intermediate storage. All shake flask campaigns were repeated in triplicate with three freshly prepared media batches.

### Spectroradiometric characterization of light exposure conditions

A portable MAS‐40 Mini‐Array Spectrophotometer (Instrument Systems, Munich, GER), was used for a detailed characterization of the illumination conditions in the experimental setup, as well as at chosen locations in the biotechnological laboratory outside the fume hood. A flexible fiber optic light guide (consisting of an EOP‐146 optical probe and the OFG‐424 fiber bundle, both by Instrument Systems) allowed measurements to be made on the interior surface of the medium bottle to check for potential filter effects mediated by the glass. Spectral data were recorded over a range from 250 to 800 nm, and analyzed using the SpecWin Light software (Instrument Systems). A standard light source emitting at isolated bands was used to check the instrument calibration.

### Shake flask cultivation of CHO cells and cell counting

An industrial CHO cell line expressing IgG was used for monitoring light‐induced effects on culture performance and productivity. For direct comparison, growth experiments were carried out in chemically defined proprietary medium (CDPM) and a commercially available standard medium (Dulbeccos' Modified Eagles Medium; DMEM; ThermoFisher, 21063029) supplemented with 10% FCS (ThermoFisher, 26140087). Cultures were established from frozen stocks and cultivated for three passages in seed train medium prior to inoculation of the main batch runs. Batch cultivations were performed in 1 L shake flasks (filling volume 250 mL), on a rotary shaker under a water‐saturated 95% air/5% CO_2_ atmosphere at 37°C. The pH was adjusted to 7.0 and the shaking conditions were set so as to avoid oxygen limitation and excessive shear stress. Viable cell count and average cell size were determined via automated image analysis with trypan blue exclusion assays (CEDEX HiRes Analyzer, Roche Life Sciences, Mannheim, GER).

### Acquisition of fluorescence spectral data

Fluorescence spectrometry of media samples was performed on a microtiter plate reader (Spark^®^ M10, TECAN Instruments, Grödig, AUT), with a sample volume of 150 μL per well in a 96 MWP (polystyrene, solid black, flat bottom, Corning Inc., USA). Sample illumination (em) and readout (ex) was performed from the top, using the built‐in monochromator optics (wavelength accuracy ex <1 nm, em < 2 nm, reproducibility <1 nm) and the internal light source reference correction. A CdSeS/ZnS alloyed quantum dot suspension (Simga‐Adrich, AUT) was used for external consistency checks. EEM spectrophotometric data were collected over a wavelength range from 300 to 500 nm (excitation) and 400 to 600 nm (emission) at a measurement interval of 10 nm (5 nm bandwidth). Samples were equilibrated to a temperature of 22 ± 1°C for analysis, and kept at this level during the entire data recording phase. For EEM scans, parallelized sample processing was restricted to meet a total maximum time of 40 min with open lid during a measurement. In controls, repeated measurements of the same sample were performed to validate that potential effects of bleaching, evaporation or adsorption of media components at the well surface are negligible over this timeframe. A matrix of ex/em pairs in the size of 20 × 20 was obtained and imported in MATLAB^®^ for further analysis and plot generation via in‐house‐written codes.

### Quantification of ROS/RNS in culture media

The level of ROS/RNS in light‐treated culture medium was determined with a commercially available assay kit that is based on the radical‐mediated conversion of a fluorogenic substrate (OxiSelect™ *In Vitro* ROS/RNS assay, CellBiolabs, USA). A protocol provided by the supplier was modified to adjust the assay conditions to the current application, which has not previously been reported in the literature. Briefly, 50 μL of sample solution were mixed with 50 μL of catalyst in a 96 WMP. After incubation for 5 min at RT, 100 μL of DCFH solution were added to each well, and fluorescence intensity (ex/em: 480/530 nm) of the solution was quantified in a multiplate reader (TECAN Instruments, Grödig, AUT). DCF solutions were used for calibration, and samples spiked with defined amounts of H_2_O_2_ were used as external standards to provide a means for comparison across platforms. The error in standard measurements was found to be <20% for samples containing <5 μmol L^−1^ H_2_O_2_, and < 10% for samples containing >5 μmol L^−1^ H_2_O_2_.

### Product titer

Secreted IgG in the culture broth was quantified using an HPLC‐based analysis protocol as used for routine quality control in manufacture. Briefly, cells were removed by centrifugation (300 *g*, 10 min), and the supernatant was diluted to an appropriate range with fresh medium. IgG molecules were isolated from other media components using a protein A column, and UV detection at 260/280 nm was used for quantification. Standard medium solutions spiked with IgG in known concentration were used for calibration, and the resultant method showed good linearity (R^2^ = 0.988) and recovery (>95%) over the relevant concentration range. IgG reference standards analyzed in triplicate showed an error of measurement of ± 12%.

### Intracellular IgG analysis

To provide an estimate of the intracellular product levels in the different culture states, a protocol was developed for immunostaining of IgG in fixed and permeabilized cells, followed by flow cytometric determination. Cell samples were taken at defined time points during the batch cultivation phase, and cells were washed by centrifugation (300 *g*, 5 min) and re‐suspension in isotonic PBS (+BSA 0.5%, EDTA 0.2 mmol L^−1^) at 4°C to remove loosely surface‐associated IgG and other proteins from the culture broth. The cell number was adjusted to 4 × 10^6^ cells mL and cells were fixed and permeabilized with a commercial sample preparation kit (Inside Stain Kit, Miltenyi Biotec, Bergisch Gladbach, GER) according to the protocol provided by the supplier. A FITC‐labeled anti‐human‐IgG‐antibody (Miltenyi Biotec, Bergisch Gladbach) was used for staining (20 min, RT) intracellular IgG molecules, and a final washing step (300 G, 5 min) in PBS (+BSA 0.5%, EDTA 0.2 m mol L^−1^) was carried out prior to single cell analysis by flow cytometry. A PARTEC Cube6 flow cytometry device (Sysmex Partec GmbH, Görlitz, GER) with standard laser/filter configuration (ex/em: 488/525 nm) was used for quantification of FITC‐stained IgG. In control experiments, a gate was defined to confine the analysis to the single cell population and exclude debris from the medium. At least 10 000 cells were collected for each measurement, and all experiments were performed in triplicate.

### Fluorescence microscopy

Images were acquired on a Zeiss Epifluorescence Axio Observer.Z1 deconvolution microscopy system (Carl Zeiss, Oberkochen, GER) equipped with LD Plan‐Neofluar objectives and the LED illumination system ‘Colibri^®^’. Exposure time and illumination parameters were kept constant to allow for direct comparison. Fresh CHO cells were drawn from the culture supernatant at the end of the exponential growth phase, washed, fixed and permeabilized as described above. Intracellular IgG was stained with the same FITC‐labeled anti‐IgG antibody as used for flow cytometric analysis. Alexa594‐WGA (5 pmoL mL^−1^; Vector Labs, Burlingame, USA) was used as a Golgi marker, and DAPI (5 μg mL^−1^, Life Technologies, Carlsbad, USA) was used to label nuclei. Single cells suspensions were transferred in cover slip‐mounted FlexiPERM^®^ culture chambers for imaging by epifluorescence microscopy.

## RESULTS

### Spectroradiometric characterization of light exposure conditions

**Figure 1:**
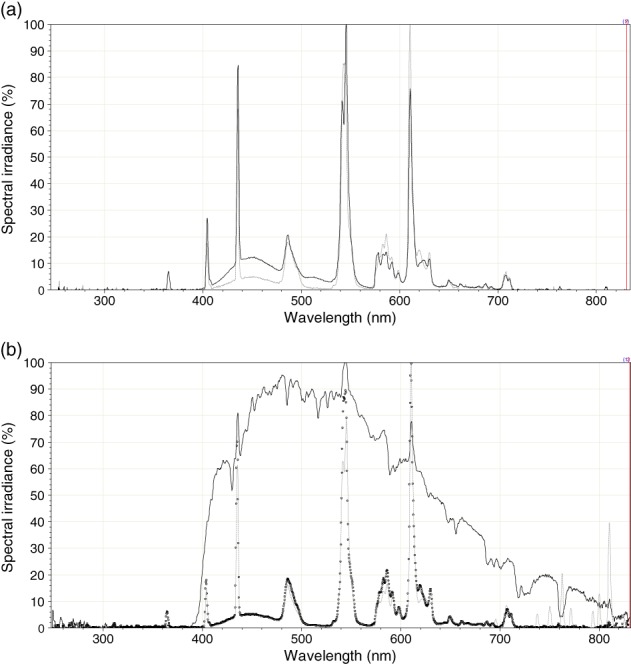
Spectroradiometric characterization of the light exposure setup for medium conditioning. (a) Relative irradiance of wavelengths emitted by the standardized light source in the experimental setup (black continuous line), compared with the illumination conditions found in a typical biosafety cabinet (grey dashed line). Note the close overlap in spectral characteristics. (b) Comparison of illumination conditions in a typical biotechnological laboratory environment. Lab corridor with artificial light only (grey dashed line), as well as in a production room at a distance of 1 m (black continuous line) and 4 m (black circles) from a window. All spectra are normalized to the maximum peak height (=100%) in each measurement.

The experimental setup for media conditioning was characterized with regard to the emitted light intensity and wavelength to provide a basis for comparison with the conditions found in development laboratories and manufacturing facilities. Spectroradiometric analysis showed a large overlap in the light quality parameters between the experimental setup and the ambient light conditions in a typical laboratory environment (Fig. 1). Importantly, the fraction of light in the UV and near‐UV region was negligible with regard to the integrated overall irradiance, which was mainly emitted in the 400–800 nm range.

In order to facilitate the detection and characterization of light‐induced effects, it was the intention in this work to mimic an environment that is on the upper limit of the irradiation intensity scale found in real‐life conditions. The overall radiometric integral in the experimental illumination setup (22.99 W m^−2^) was approximately five‐fold higher than that found under standard lighting situations at various points in the laboratory (e.g. 4.04 W m^−2^ in a standard biosafety cabinet), but can easily be reached at locations close to windows. However, the onset and evolvement of light‐induced changes under real‐life working conditions may be somewhat delayed compared with the more extreme conditions in this light stress test.

### Spectral analysis of media photo‐degradation

Excitation/emission matrix (EEM) spectroscopy is a powerful method to rapidly assess qualitative and quantitative changes in complex mixtures of biological material, such as cell culture media.^5^, ^12^ Due to the measurement principle, EEM spectroscopy is intrinsically focused on photophysically active molecules, and offers high sensitivity and throughput capability. These properties render it ideally suited for detecting and monitoring light‐induced changes in media composition, and also allow for easy method transfer to routine quality control applications in industrial biotechnological production. In order to best possibly avoid inner filter effects and work above the cutoff for high absorbance of interfering components, the EEM wavelength region investigated in this study was limited to the range 300–500 nm (λ_ex_) and 400–600 nm (λ_em_).^12^, ^13^

**Figure 2:**
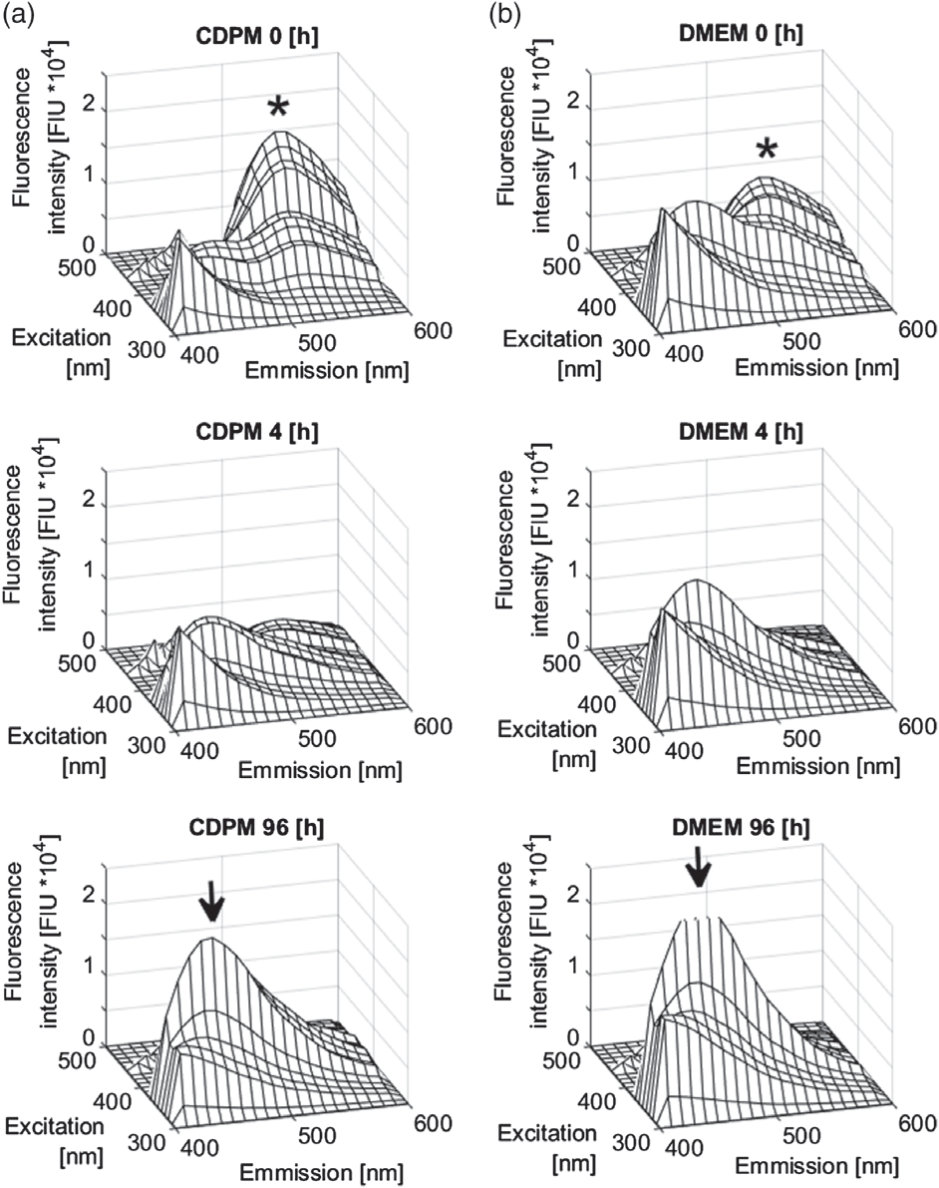
Assessment of media photo‐degradation using EEM. EEM landscape plots generated from (a) CDPM and (b) DMEM after a light exposure time of 0, 4 and 96 h, respectively. In the upper and lower panels, the wavelengths bands assigned to the cluster1 and cluster2 region are marked at the point of highest intensity (cluster1: asterisks, cluster2: arrows).

Contrasting with the dark control, the EEM landscape plots of light‐exposed CDPM and DMEM showed pronounced topographical changes over time, with both regions of increasing and decreasing fluorescence intensity (Fig. 2). Despite the overlapping effect of multiple different fluorophores, a clear differentiation could be made between specific regions of interest. Most notably, both media showed a rapid decrease in the band around 450/540 nm (λ_ex_/λ_em_) and the gradual formation of a new fluorescence band at around 360/450 nm (λ_ex_/λ_em_), which were for the following experiments designated ‘cluster 1’ and ‘cluster 2’, respectively.

**Figure 3:**
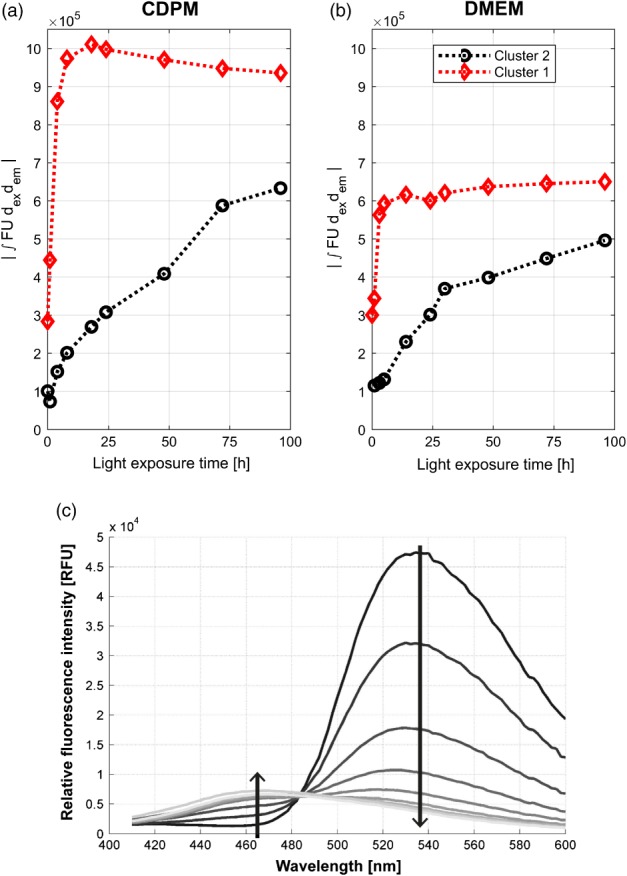
Kinetics of spectral shifts in test media and buffered riboflavin solution. Formation/degradation kinetics of the cluster 1/cluster 2 region in EEM landscape plots of (a) CDPM and (b) DMEM. (c) Spectral change in Rf fluorescence properties (5 µ mol L^−1^ solution in isotonic PBS pH 7.1) upon light irradiation under the standardized experimental setup. Spectra were recorded at hourly intervals over a total light exposure time of 0–8 h.

Spectral data and kinetics of alteration strongly suggested that Rf is the principle component visible in the cluster 1 region. Additional control experiments with Rf standard solutions in PBS (Fig. 3(c)) confirmed this assumption. The main fluorescent component was nearly completely degraded after a LET of about 10 h in CDPM, and on an even faster time scale (∼5 h) in DMEM (Fig. 3(a), (b)). Up to concentrations of 5 μ mol L^−1^ in Rf standard solutions, the emission at 540 nm was reduced by more than 90% within 8 h of light exposure. The isolated fluorescence spectra recorded at λ_ex_ = 366 nm for pure Rf in buffer also showed a gradual increase in the emission band around ∼465 nm, which correlated with the emission decrease at ∼540 nm.

While it was not the aim of this study to identify the exact species and formation pathways of the degradation products obtained under the given conditions, the spectral data corresponded well to the photophysical properties of lumichrome (LmC), one the most abundant Rf derivatives.^14^ The results in this regard match other reports on light‐induced formation of LmC from Rf, and it is likely that progressive LmC formation also is the principal cause for characteristic intensity changes in the cluster 2 region (λ_ex_/λ_em_: 360/450 nm).^5^

However, direct comparison with the degradation process of pure Rf in buffer suggested that also other species contribute to the observed fluorescence pattern. The relative signal increase at band 360/450 nm (λ_ex_/λ_em_) in pure Rf solutions was far lower than the increase observed in media (Fig. 3(a), (b)). Also, while Rf degradation in cluster 1 seemed to be completed after no more than 10 h, the main fluorescence band of cluster 2 continued to increase over a far longer period of LET (Fig. 3(a), (b)). This points to one or several additionally contributing species that either are photophysically active themselves or can indirectly affect other fluorescent components via inner filter effects or by altering photosenitized reaction pathways.^5^

### Light‐induced radical formation

It is well known that the light‐induced degradation of Rf in interplay with Trp and Tyr can trigger the formation of toxic radicals, including hydrogen peroxide and singlet oxygen species.^5^, ^6^, ^7^, ^8^, ^15^, ^16^ In order to test whether the photosensitized reactions of Rf in CDPM and DMEM induce a higher level of ROS/RNS, the total radical burden in media samples was determined by fluorimetric assay. The DCFH‐based method was chosen based on its ability to provide an overall index of the total oxidative stress, and good potential for later transferability to on‐site applications.^17^ However, it should be emphasized that the non‐selective measurement principle does not allow detection of the specific type(s) of radicals involved in the process.

**Figure 4:**
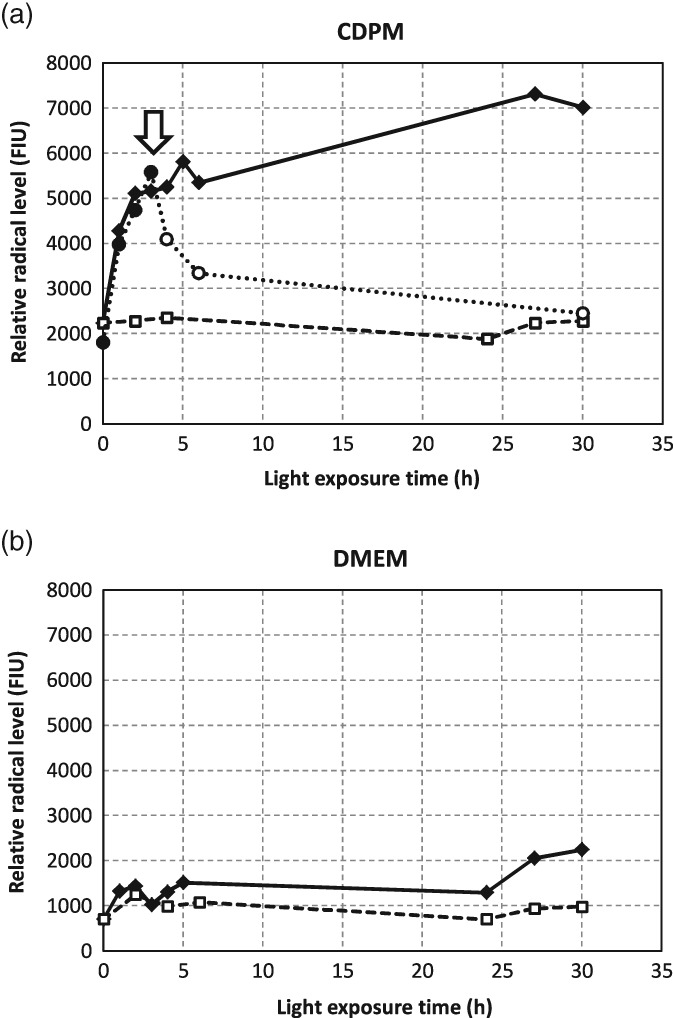
Light‐induced ROS/RNS formation and decay in culture media. Relative ROS/RNS activity in (a) CDPM and (b) DMEM exposed to the artificial light source (continuous line with closed symbols) or stored under similar conditions in the dark (dashed line with open symbols). For each sample, the relative radical level was quantified indirectly using the DCFH conversion rate. Time‐dependent radical decay in irradiated CDPM was assessed in a pulse‐chase experiment by exposing the sample to light for a total duration of 3 h, followed by a subsequent dark phase (dotted line in plot (a)). The switch to dark conditions is indicated by an arrow (plot (a)).

A pronounced increase in ROS/RNS formation was found for CDPM, where, following a drastic increase in the early phase of light exposure, a stable level about three‐fold higher compared with the initial value was reached (Fig. 4(a)). No significant change was observed for the dark control sample. The ROS/RNS burden in DMEM differed substantially from that in CDPM. The basal radical level in the dark control was significantly lower, and only a slight increase was visible upon light irradiation (Fig. 4(b)). Experiments with additional Rf spiking (see Supporting information) showed no changes in the radical level, suggesting that in the present case, Rf photosensitization was not the main factor for substantially higher oxidative stress under light.

To investigate in further detail the time course of the light‐induced ROS/RNS stress response, and determine how fast elevated radical levels may decline again, pulse‐chase experiments were performed with CDPM being first exposed to light and then monitored throughout a subsequent dark phase. The increase in ROS/RNS activity was found to be gradually reversible after returning to dark conditions, and after ∼24 h had reached the initial basal level (Fig. 4(a), dotted line). In calibration assays using H_2_O_2_ as a standard, the basal (dark control) radical level in CDPM and DMEM corresponded to ∼3 μmol L^−1^ and ∼1 μmol L^−1^ of H_2_O_2_ in PBS, respectively.

### Light‐induced effects on culture physiology

The interlinks between light irradiation and culture physiology were investigated in shake flask cultivations of CHO cells in pre‐conditioned CDPM and DMEM. This experimental strategy allowed focusing on the effect of photo‐chemical reactions in the media, while excluding any detrimental impact caused by the direct exposure of cells to light.

**Figure 5:**
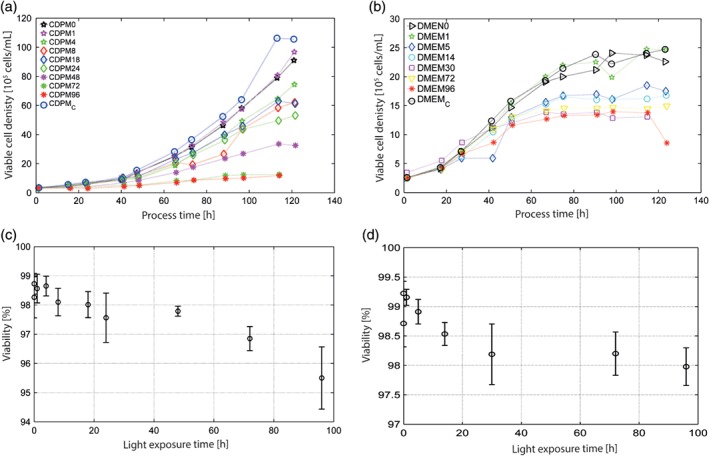
Culture physiology in photo‐damaged media. Growth characteristics of CHO shake flask cultures in (a) CDPM and (b) DMEM pre‐conditioned by light irradiation for 0–96 h. Average CHO cell viability depending on total light irradiation time in (c) CDPM and (d) DMEM.

Pronounced effects were observed on growth characteristics and viability depending on the light exposure profile. In CDPM, an irradiation phase of 1 h resulted in only a minor decrease in VCC compared with the controls, but ample shifts were visible in media exposed to light for 4 h or longer (Fig. 5(a)). VCC_max_ was reduced by 18%, 35% and 57% after 4, 24 and 48 h of medium irradiation, respectively. Cell growth was nearly completely inhibited in CDPM after a LET of 72 h or longer. As illustrated in Fig. 5(c), the average cell viability remained on a comparatively high level even for prolonged irradiation times, with a small but statistically significant decrease to ∼95% at maximum LET.

In commercial DMEM, which was not optimized for the industrial CHO cell line used in this study, generally lower final cell concentrations were obtained. Also, the transition from the exponential to the stationary growth phase occurred earlier, at a process time of about 80 h (Fig. 5(b)). DMEM light exposure for 4 and 30 h resulted in a decrease of VCC_max_ by 16% and 30%, which is in good correspondence with the relative decrease observed in CDPM. However, no further decrease in VCC was visible beyond that point (72, 96 h of light exposure). Most probably, the inferior growth potential provided by the non‐optimized media formulation, in combination with 30 h of photo‐degradation, had already compromised proliferation capacity to an extent that obscured any further downward shifts. Interestingly, also cells in preconditioned DMEM retained a high average viability over the entire range of LET. Control measurements of LDH in the culture supernatant showed no signs of increased cell lysis in both media (data not shown).

### Morphological changes in photo‐degraded media

**Figure 6:**
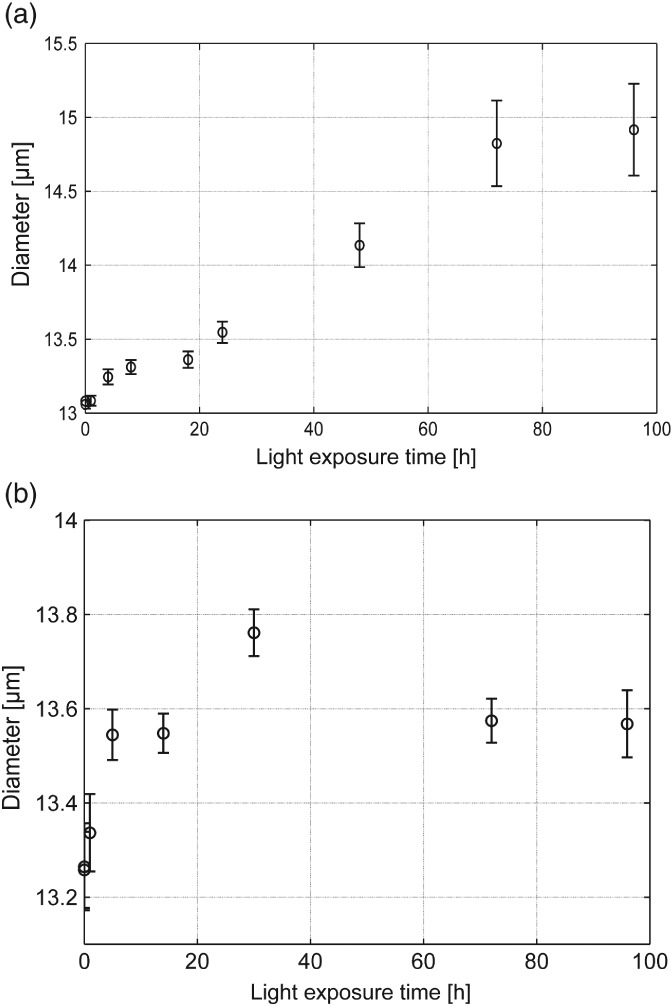
Morphological changes in light‐irradiated medium. Average cell diameter in CHO shake flask cultures in light‐treated (a) CDPM and (b) DMEM depending on the total irradiation time.

A notable effect on average cell size was visible in dependence of irradiation time. The change was more pronounced in the case of the CDPM, ranging from ∼13.1 ± 0.04 μm in non‐treated medium to ∼15.0 ± 0.3 μm after a LET of 96 h (Fig. 6(a)). In DMEM, the mean increase in cell diameter was smaller but still significant (13.3 ± 0.1 to 13.7 ± 0.05 μm). Similar to VCC, no further change was observed by extending the LET of DMEM beyond 30 h (Fig. 6(b)).

### Impact of media photo‐degradation on product formation

In principle, the reasons for lower specific product titers may either lie in a decreased IgG production rate (e.g. caused by alterations in protein expression, post‐translational modification and secretion) or in an increased IgG degradation rate in the irradiated medium (e.g. due to higher oxidative stress levels or direct IgG photolysis).^10^, ^18^, ^19^ In this study, a bimodal analytical approach was taken to gain information on the extracellular, as well as on the intracellular IgG concentration.

**Figure 7:**
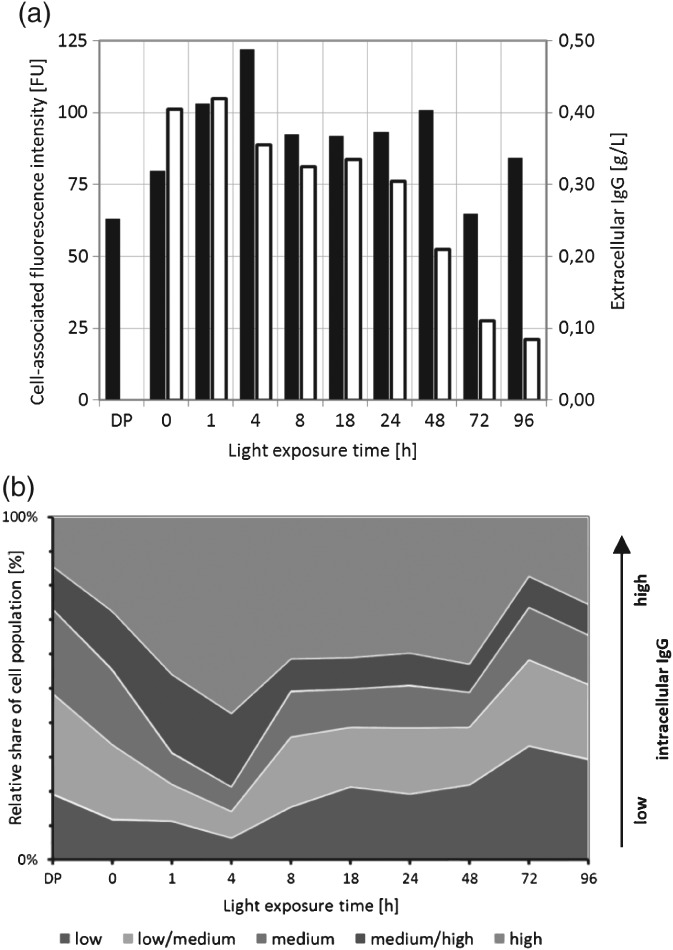
Analysis of IgG formation in CHO shake flask cultures in light‐irradiated CDPM. (a) Intracellular IgG was detected via fluorescence‐immunostaining in fixed and permeized cells with subsequent flow cytometric analysis (mean cell‐associated fluorescence intensity; closed bars), extracellular IgG was quantified by HPLC (open bars). (b) Proportional composition of the CHO cell population at different light exposure times. For each time point, cells were stratified according to the intracellular IgG level (high/medium‐high/medium/medium‐low/low intracellular IgG staining).

IgG levels in the supernatant were determined using a HPLC method for routine quality control. A substantial, gradual loss in productivity with increasing LET was found (Fig. 7(a)). At a total irradiation time of 96 h, only ∼20% of the titer without medium pre‐incubation was obtained. The stepwise decrease in the final IgG concentration directly correlated with the decrease in VCC (cf. Fig. 5), suggesting that the main cause for lower overall productivity is a reduced biomass concentration (or, in other words, qP is constant). Accordingly, it would be expected that the protein synthesis machinery is still working regularly and is not negatively affected by media photo‐degradation. A set of analyses conducted in the framework of routine quality control of the IgG product (e.g. charge variant profiling) showed no indications for alterations in performance as a consequence of media irradiation. This indicates that the IgG secreted by cultures in photo‐degraded media was lower in amount, but otherwise unimpaired.

Flow cytometric analysis revealed some marked changes in the intracellular IgG pool over LET. Progressive IgG accumulation was observed in cells grown in irradiated medium up to a total LET of 4 h (Fig. 7(a)). In the segregated analysis of the culture population, this accumulation process was reflected by a pronounced shift (up to 57.3% at 4 h LET) to the high‐IgG cell class (Fig. 7(b)). Interestingly, comparatively minor alterations were observed in the class of cells with low IgG content over the same LET range (11.6% to 6.3%, for a LET from 0 to 4 h, respectively). Beginning with a LET >4 h, the proportional share of cells with low and low/medium IgG content increased steadily, and only 25.4% of cells were still in the high‐IgG class when grown in heavily photo‐damaged medium (LET 96 h). Despite these clear shifts in the population composition, the mean intracellular IgG level (Fig. 7(a), closed bars) remained on a comparatively stable level between a LET of 8 to 96 h.

### Intracellular IgG localization

**Figure 8:**
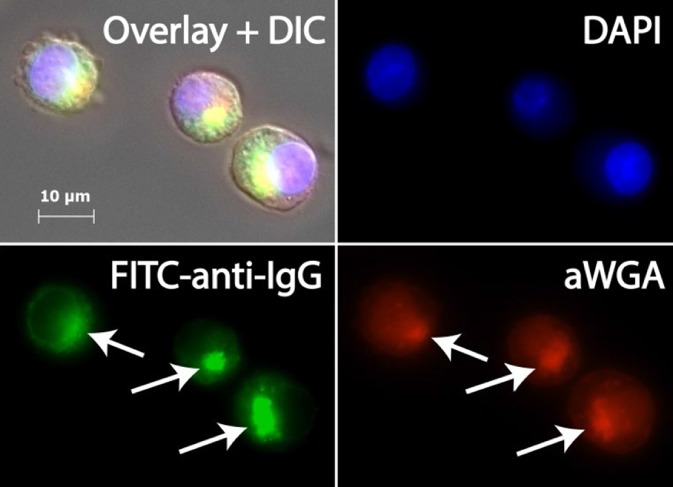
Fluorescence micrograph of intracellular IgG localization in CHO cells. Cells from shake flask cultivation (0 h LET, end of exponential phase) were fixed, permeized, and stained with the FITC‐labeled anti‐IgG antibody (FITC‐anti‐IgG). Alexa594‐conjugated wheat germ agglutinin (aWGA) was used as a Golgi marker, and nuclei were labeled with a DNA‐specific stain (DAPI). Note the high degree of co‐localization between IgG and Golgi compartments (arrows), which is clearly visible in the morphological overlay with differential interference contrast (Overlay + DIC).

Image analysis by deconvolution fluorescence microscopy provided further insights on the intracellular localization of the IgG product. A basal amount of IgG was found distributed homogenously across the entire cytoplasmatic space (Fig. 8). Most cells additionally exhibited a contoured region with substantially higher IgG concentration, located unipolarly on one side of the nucleus. This points to the ER compartment and the Golgi apparatus as the main sites for IgG accumulation, which is in accordance with the canonical pathway. A high rate of co‐localization between the Anti‐IgG antibody and the Golgi marker wheat germ agglutinin substantiated this assumption.^20^ Despite the shifts in other culture parameters, no marked difference in the spatial arrangement of IgG accumulation sites was observed in cells grown in light‐irradiated medium (data not shown).

## DISCUSSION

The central aim of this work was to establish a link between the process of photosensitized media degradation and culture performance. For this it was first necessary to select and characterize adequate light conditions with view on practical relevance. It was reported that the spectral composition of the incident light is a critical determinant for the viability of biotechnological cultures exposed to direct illumination.^21^ Indirect effects, caused by *a priori* light exposure of the culture medium, will most probably exhibit the same, wavelength‐dependent variations, as different chemical groups will be differentially affected at a given wavelength. While a detailed investigation of the individual impact of certain wavelengths was not within the scope of this study, it was nevertheless possible to derive some interesting general conclusions. The low fraction of light in the <400 nm range (cf. Fig. 1(a)) suggests that the observed effects are not related to UV irradiation, which may seem likely at first glance. This finding is in agreement to previous studies, who demonstrated the same.^5^ Also, it can be expected that the observed degradation effects will apply in similar fashion to a wide range of translucent bioreactors or vessels, since alterations in the inherent filter effect of different glass or plastic materials mainly affects the UV part of the spectrum.

Based on the close spectral overlap between the light used in the experimental setup and the light conditions in typical laboratory environments, we expect the same molecular components to be involved in both cases. It should be noted that this may not apply for workspaces that are exposed to natural light (e.g. workbenches close to windows), which feature a continuous sunlight spectrum and several‐fold higher radiometric integrals (Fig. 1(b)). As an important side aspect, this study wants to direct further attention to light quality and illumination conditions in production or research facilities. Not only in the context of creating pleasant working environments, but also as a potential biasing factor that should be considered in site audits and risk assessment processes.

The evaluation of EEM landscape plots in comparison with available data in literature pointed to a major role for Rf and the corresponding degradation product lumichrome (LmC) in the light‐triggered reaction cascade. Rf is contained in most culture media in typical concentrations up to 1.5 µmol L^−1^, and has a key function in oxygen reduction, energy production, vitamin metabolism and other antioxidative protection mechanisms.^22^ It is one of the few well‐described examples of media components that are prone to photolysis with proven impact on culture performance.^4^, ^8^, ^23^, ^24^ However, owing to an exceptionally high quantum yield, Rf could eventually mask other constituents in the EEM spectrum, which should be considered for interpretation.^12^

In general, it should be noted that the relationship between photophysical properties and underlying compositional changes in media may not always be straightforward. Certain adverse effects on cell physiology or culture performance may be directly related to the concentration of a given fluorescent compound, and hence to the signal intensity in the EEM plot (e.g. the depletion of Rf). Other media quality factors, in contrast, may only partially or not at all be connected to shifts in the spectral properties (e.g. the formation of ROS/RNS). For the latter case without direct mechanistic linkage between compound degradation and spectral changes (but reasonable correlation), EEM analysis may still provide a useful ‘soft sensor signal’ to establish methods for media quality monitoring.

In this study, comparatively similar alterations were observed in the EEM landscape plots of two substantially different media formulations (one chemically defined and one serum‐supplemented medium). It is thus hypothesized that also the underlying mechanistic processes may be of similar nature. On this basis, it may be possible to develop a generic monitoring protocol (after ‘fine calibration’ to the specific mixture) to assess media photo‐damage in different bioprocesses.

Contrasting with earlier studies with HEPES‐supplemented RPMI and normal DMEM‐F12 medium, which identified Rf photo‐conversion as the main causative factor for ROS/RNS formation, this present study data do not support such a strict mechanistic link.^15^, ^25^ According to EEM spectra (Fig. 3(a), (b)), Rf was completely degraded after no more than ∼10 h, but total radical activity remained on a high level after this point. Results also show that the basal level of radical species in cell culture media must not necessarily be close to zero.^7^, ^26^ A constant amount of radicals seems to be formed *in situ* without involvement of exogenous stress factors such as light, and to a different extent in different media formulations. Alternatively, it is possible that serum components (as were supplemented to DMEM) act as radical scavengers, which was reported for similar systems.^16^, ^25^

In this study, the predominant effect of media photo‐degradation on culture physiology seemed to be the transition to a steady state with partial to near‐complete growth arrest, but without ample induction of apoptosis or lysis.

Interestingly, the gradual decrease in growth potential in CDPM continued for LETs beyond 10 h, which was the presumed point of complete Rf depletion (cf. Fig. 3(a), cluster1). This indicates that also other photosensitive compounds, possibly fluorescent species in the cluster 2 region of the EEM plot, contribute to the observed effect. Based on the finding that the decrease in VCC with prolonged LET did not closely correlate with the increase in total radical levels (see Fig. 4), it is concluded that ROS/RNS may not be the main factor responsible for growth impairment. This assumption is further supported by the fact that pronounced growth‐inhibiting effects were also observed in serum‐supplemented DMEM, which had only slightly elevated radical levels under light exposure. Still, a combined effect (e.g. caused by radicals breaking down other vital components) cannot be excluded.

Based on the close correlation between cell diameter increase and the decrease in growth potential (Figs 5 and 6), it is likely that the higher cell volume in this case was mainly a result of lower mitotic activity. Alternatively, it is possible that an increased uptake of osmotically active compounds, e.g. amino acids or other nutrients, induced a secondary swelling process. Such up‐regulation of amino acid transporters is a well‐known response of mammalian cells to oxidative stress, and should hence be more pronounced in CDPM than in DMEM, as found in the results obtained.^27^

Productivity data suggested that the overall reduction in cell concentration is the main limiting factor in photo‐damaged medium, as was reported elsewhere.^28^ However, it was found that additionally the IgG processing machinery seems to be altered in light‐irradiated medium, and the ‘intracellular titer’ is somewhat decoupled from the extracellular concentration. These differences between intracellular IgG level and overall qP imply a newly emerged bottleneck in the antibody maturation chain or secretion pathway. Such dynamic transitions between different rate‐limiting steps in the IgG production cascade, with pronounced intracellular accumulation, have been observed previously in response to changed feeding conditions.^29^

It should be noted in this regard that the immunofluorescence‐based analysis protocol provides only a rough estimate of the IgG concentration in a cell at a given time point, but does not allow for discriminating between increased expression and reduced secretion rates. Also, it is not exactly known which steps in the IgG biosynthesis and maturation process (e.g. chain assembly and further posttranslational modifications) influence the binding of the anti‐IgG antibody. In microscopic image analysis, no signs for accumulation of IgG‐positive secretory vesicles were found, suggesting that any light‐induced impact on the IgG processing machinery primarily affects stages prior to the final membrane fusion step. One potential explanation would be a retention of unassembled or incompletely glycosylated IgG chains in the ER and Golgi, as reported elsewhere.^30^ While further mechanistic details remain to be elucidated, this present study underlines the role of flow cytometric *in situ* analysis as an effective strategy to reveal changes in the IgG production/secretion cascade that are not visible by merely checking the overall titer profile (qP). Today, advanced methods to monitor productivity in a single‐cell segregated fashion are available that merit broader implementation in process optimization and control.^31^

Overall, this study adds further support to the still underestimated role of – accidental or knowingly tolerated – light‐mediated effects on culture performance in biotechnological manufacture. Exposure to ambient light for only a limited amount of time may severely compromise growth and productivity. It is highly advisable that appropriate monitoring strategies be implemented in the routine quality control and risk assessment protocols, especially when working with transparent bioreactors or disposable systems.

## CONFLICT‐OF‐INTEREST STATEMENT

The authors declare no commercial or financial conflict of interest.

## Supporting information

Supporting Information A supplemental information file containing additional data on DMEM specifications, spike experiments with Rf in DMEM and a multivariate analysis of the key variables of the CDPM experiments can be downloaded from:
**Figure S1.** Multivariate analysis of key variables from CDPM experiments byPCA using two principle components. Score, loading and cumulated R2 and Q values are given for a dataset of 16 observations corresponding to different media light exposure times from 0 to 96 h.
