# Supplementary material for: Media photo‐degradation in pharmaceutical biotechnology – impact of ambient light on media quality, cell physiology, and IgG production in CHO cultures
Source: J Chem Technol Biotechnol. 2018 Jun 1;93(8):2141–51. doi: 10.1002/jctb.5643 (PMC6055871; doi:10.1002/jctb.5643)
Supplement: Supplementary file 1 — Supporting Information A supplemental information file containing additional data on DMEM specifications, spike experiments with Rf in DMEM and a multivariate analysis of the key variables of the CDPM experiments can be downloaded from: Figure S1. Multivariate analysis of key variables from CDPM experiments byPCA using two principle components. Score, loading and cumulated R2 and Q values are given for a dataset of 16 observations corresponding to different media light exposure times from 0 to 96 h. [file JCTB-93-2141-s001.docx]

**Supplemental Information to research manuscript**

**Media photo-degradation in pharmaceutical biotechnology – Impact of ambient light on media quality, cell physiology, and IgG production in CHO cultures**

1. **DMEM specifications**

For comparative analysis in a well-defined, commercially available medium, experiments were repeated in DMEM (Dulbeccos’ Modified Eagles Medium; DMEM; ThermoFisher, 21063029) supplemented with 10 % FCS (ThermoFisher, 26140087). The detailed composition of this medium is given in the table below.

| Glycine | 30.00 |
| --- | --- |
| L-Arginine hydrochloride | 84.00 |
| L-Cystine-2HCl | 63.00 |
| L-Glutamine | 584.00 |
| L-Histidine hydrochloride-H2O | 42.00 |
| L-Isoleucine | 105.00 |
| L-Leucine | 105.00 |
| L-Lysine hydrochloride | 146.00 |
| L-Methionine | 30.00 |
| L-Phenylalanine | 66.00 |
| L-Serine | 42.00 |
| L-Threonine | 95.00 |
| L-Tryptophan | 16.00 |
| L-Tyrosine disodium salt dihydrate | 104.00 |
| L-Valine | 94.00 |
| Choline chloride | 4.00 |
| D-Calcium pantothenate | 4.00 |
| Folic acid | 4.00 |
| i-Inositol | 7.20 |
| Niacinamide | 4.00 |
| Pyridoxine hydrochloride | 4.00 |
| Riboflavin | 0.40 |
| Thiamine hydrochloride | 4.00 |
| Calcium chloride (CaCl2) (anhyd.) | 200.00 |
| Ferric nitrate (Fe(NO3)3-9H2O) | 0.10 |
| Magnesium sulfate (MgSO4) (anhyd.) | 97.67 |
| Potassium chloride (KCl) | 400.00 |
| Sodium bicarbonate (NaHCO3) | 3700.00 |
| Sodium chloride (NaCl) | 4750.00 |
| Sodium phosphate monobasic (NaH2PO4-H2O) | 125.00 |
| D-Glucose (dextrose) | 4500.00 |
| HEPES | 5958.00 |

1. **Additional control experiments with RF standard solutions**

500 ml of a solution of 5 µM riboflavin in isotonic PBS (pH 7.1) were placed in Duran® glas bottles at an orbital shaker with mild agitation, and irradiated over a total duration of 8h at room temperature (22 °C) with the same standard light sources used for media degradation experiments (4x E27 energy-saving daylight bulbs; 20W, 6500K, 1215 lm). Sample aliquots (1 ml) were taken at hourly intervals and immediately analysed for fluorescent properties via EEM scans (see materials & methods section). The decrease in the riboflavin-associated fluorescence cluster (λ_em_ = 366 nm, λ_em_ ~ 540 nm; see figure 3c for the isolated signal line at this wavelength over time) corresponded closely to the shift observed in media for the same wavelength region. Via the same protocol, solutions of 0.5 µM and 1.5 µM riboflavin were tested, with an even faster degradation time course. The concentrations tested were chosen with reference to the most common concentration level in cell culture media.

1. **Multivariate analysis of key variables in the CDPM experiments**

A multivariate analysis of the key variables from the CDPM dataset was conducted in the SIMCA14 software suite provided by Umetrics (Sartorius-Stedim, SWE). The investigated variables included EEM cluster1 / cluster2 spectral changes (expressed as the difference in fluorescence intensity towards the starting point), ROS/RNS level, cell concentration, viability, average cell diameter, IgG titer and intracellular IgG accumulation. All data were normalized by unit variance scaling prior to principle component analysis with a non-hierarchical model, using different numbers of components.

Corresponding to the faster kinetics of fluorescence changes in the wavelength region of ‘cluster 1’, samples taken after shorter light exposure times are in the model more closely associated to this spectral shift. The decreasing fluorescence in cluster 1 correlates well to the ‘physiological’ impairments of the culture (slow growth, low viability, low IgG titer, and – inversely – to cell diameter). Wavelength cluster 2 (gradually building up with longer irradiation times) seems to be closely associated to the increase in radical burden. The second component (inclusion of more than two components did not increase the model quality further) is governed by the inverse relationship between EEM cluster 1 fluorescence loss and intracellular accumulation of IgG.


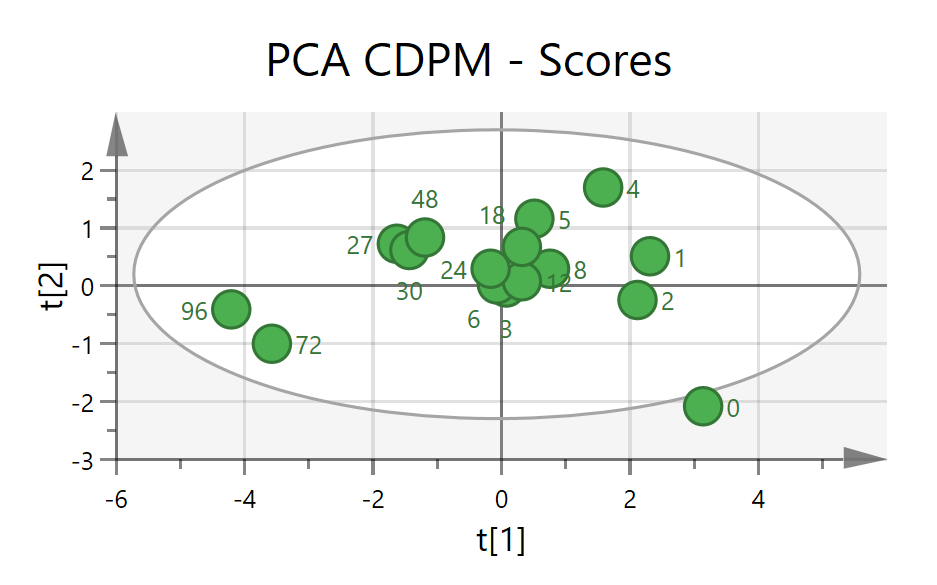


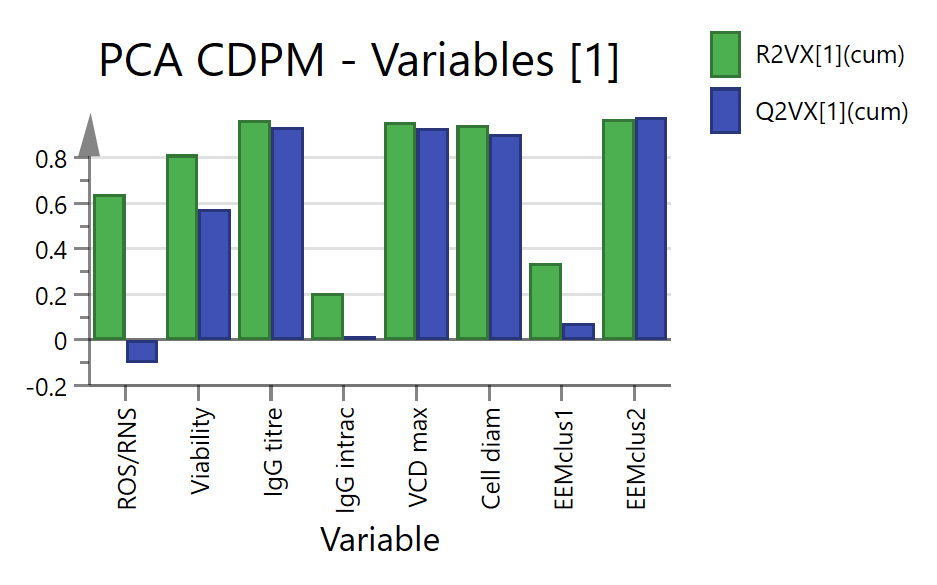


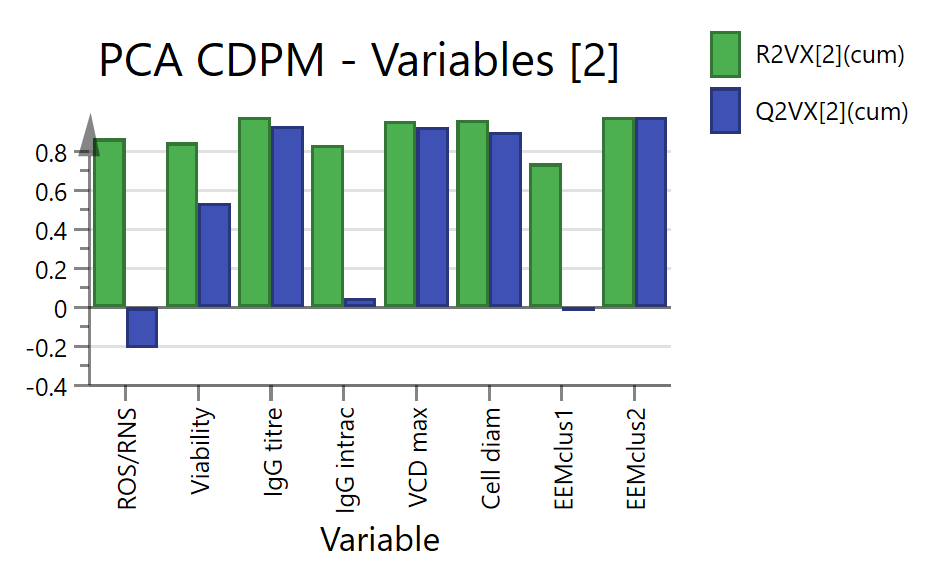

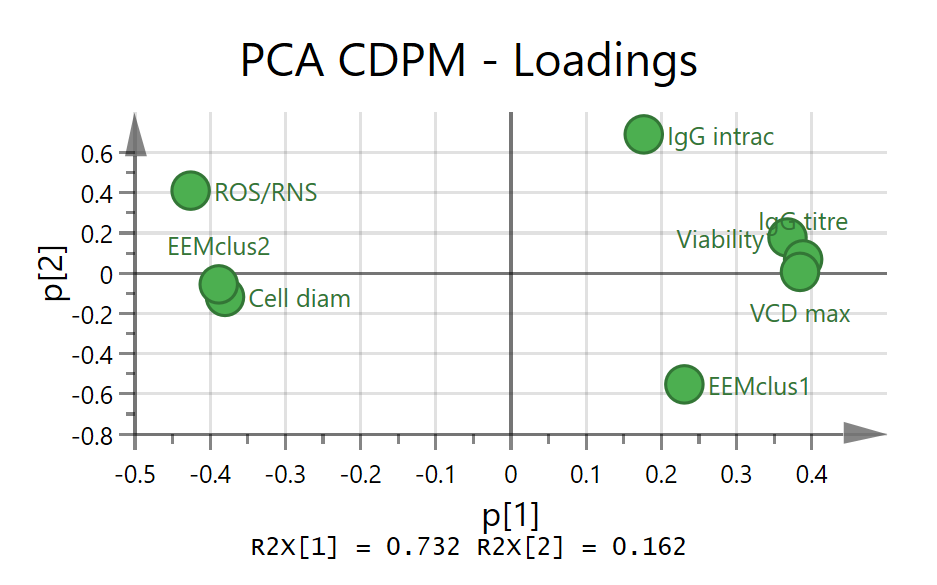


Figure S1: Multivariate analysis of key variables from CDPM experiments via PCA using two principle components. Score, loading and cumulated R2 and Q values are given for a dataset of 16 observations corresponding to different media light exposure times from 0 – 96h.
